# 

**Published:** 1894-07-28

**Authors:** 


PRICE TWOPENCE.
No. 409, Vol. XVI.—Ju y 28th, 1894.
[Registered at the General Post Office as a Newspaper for transmission in the United Kingdom and Abroad.]

WITH EXTRA NURSING SUPPLEMENT.
**Per Annum, 10s. 6d., post free.**
**Monthly Parts, 6d.; post free, 8d.**
**Ditto per Annum, 8s. 6d., post free.**

### THE HOSPITAL

LIBRARY

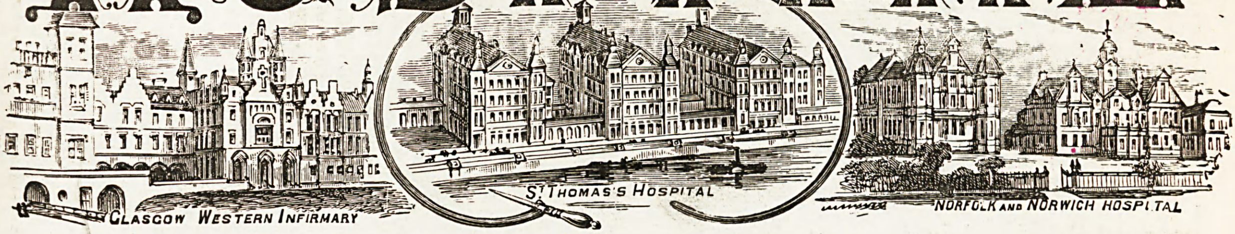

No. 409. Vol. XVI. **WITH EXTRA NURSING SUPPLEMENT.** Saturday, July 28th, 1894.

